# Altered Subcortical Brain Volume and Cortical Thickness Related to Insulin Resistance in Type 2 Diabetes Mellitus

**DOI:** 10.1002/brb3.70055

**Published:** 2024-10-04

**Authors:** Zidong Cao, Limin Ge, Weiye Lu, Kui Zhao, Yuna Chen, Zhizhong Sun, Wenbin Qiu, Xiaomei Yue, Yifan Li, Shijun Qiu

**Affiliations:** ^1^ First Clinical Medical College Guangzhou University of Chinese Medicin e Guangzhou China; ^2^ Department of Endocrinology The First Affiliated Hospital of Guangzhou University of Chinese Medicine Guangzhou China; ^3^ Department of Radiology The First Affiliated Hospital of Guangzhou University of Chinese Medicine Guangzhou China; ^4^ State Key Laboratory of Traditional Chinese Medicine Syndrome Guangzhou China

**Keywords:** brain volume, cortical thickness, insulin resistance, structural magnetic resonance imaging, Type 2 diabetes mellitus

## Abstract

**Purpose:**

The objective of this study is to examine the alterations in subcortical brain volume and cortical thickness among individuals diagnosed with Type 2 diabetes mellitus (T2DM) through the application of morphometry techniques and, additionally, to investigate the potential association between these modifications and insulin resistance (IR).

**Materials and methods:**

The present cross‐sectional study comprised a total of 121 participants (*n* = 48 with healthy controls [HCs] and *n* = 73 with T2DM) who were recruited and underwent a battery of cognitive testing and structural magnetic resonance imaging (MRI). FreeSurfer was used to process the MRI data. Analysis of covariance compared discrepancies in cortical thickness and subcortical brain volume between T2DM and HCs, adjusting for the potential confounding effects of gender, age, education, and body mass index (BMI). Exploratory partial correlations investigated links between IR and brain structure in T2DM participants.

**Results:**

Compared with HCs, individuals with T2DM demonstrated a cortical thickness decrease in the right caudal middle frontal gyrus, right pars opercularis, left precentral gyrus, and bilateral superior frontal gyrus. Furthermore, this study for T2DM found that the severity of IR was inversely related to the volume of the left putamen and left hippocampus, as well as the thickness of the left pars orbitalis, left pericalcarine, right entorhinal area, and right rostral anterior cingulate gyrus.

**Conclusion:**

The evidence for structural brain changes in T2DM was observed, and alterations in cortical thickness were concentrated in the frontal lobes. Correlations between IR and frontal cortical thinning may serve as a potential neuroimaging marker of T2DM and lead to various diabetes‐related brain complications.

## Graphical abstract

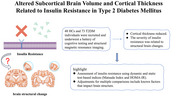

## 1 Introduction

Diabetes mellitus (DM) constitutes a rapidly expanding global public health emergency of the 21st century, and it was estimated that 537 million individuals were afflicted worldwide in 2021 (International Diabetes Federation 2021). The majority of diabetes cases, more than 90%, are classified as Type 2 DM (T2DM) (Zheng, Ley, and Hu et al. 2018). It is a disease mainly characterized by elevated blood glucose due to relatively insufficient insulin secretion. Insulin resistance (IR), described as diminished responsiveness to insulin's effects, is the iconic feature of T2DM. Insulin is an endocrine peptide hormone essential for controlling glucose homeostasis. In addition to its peripheral effects, there has been a growing recognition of its multidimensional role in the central, including cognitive function, depression, and food intake (Agrawal et al. 2021). Both peripheral and central IR might have a detrimental impact on the brain, such as a deterioration in memory, mood, and cognition, as well as the manifestation of symptoms resembling anhedonia (Sripetchwandee et al. 2018). Various neuroimaging evidence also suggests that IR may cause cognitive impairment and brain abnormalities (Cui et al. 2022).

Advances in brain image processing and analysis have allowed us to quantitatively measure structural indicators, like cortical thickness and volume, in each anatomical region on the basis of brain magnetic resonance imaging (MRI). In T2DM patients, meta‐analytic research on structural regional cerebral impacts showed a reduction in overall brain as well as a drop in the volume of the orbitofrontal, occipital hippocampus, and basal ganglia (Moulton et al. 2015). The Maastricht study also revealed that T2DM was linked to hippocampal subregions atrophy, independently of lifestyle and cardiovascular risk factors (Monereo‐Sánchez et al. 2023). T2DM may exacerbate atrophy of specific gray matter regions (Li et al. 2020); a major case–control study in T2DM has found cortical atrophy mirrors patterns seen in Alzheimer's disease (Moran et al. 2013). Other investigations have reported the presence of deficiencies in cortical thickness and surface area that are dispersed diffusely over the brain (Brundel et al. 2010; Peng et al. 2015). Alterations in subcortical brain volume and cortical thickness correlate with common features among T2DM patients. Investigating these alterations could offer insight into the neurobiological underpinnings of T2DM. It is imperative to conduct additional research on brain structure in order to enhance comprehension of the mechanisms linked to this disease and aid in the advancement of neurobiologically informed treatments.

To date, investigations of the brain structure of individuals with T2DM have faced limitations due to the presence of heterogeneity in the adjustment for multiple comparisons and the inconsistent inclusion of factors that are known to impact brain structure. In particular, cortical thickness has been demonstrated to decrease with age (Frangou et al. 2022; Masouleh et al. 2019) and body mass index (BMI) (Masouleh et al. 2019). Therefore, it is crucial to take these variables into account while conducting analyses. Moreover, prior research endeavors have exhibited less emphasis on the investigation of IR. The extent to which IR is accountable for the observed impacts of T2DM on brain anatomy remains uncertain at this time.

In this work, we undertook a cross‐sectional study of subcortical brain volume and cortical thickness in individuals suffering from T2DM and performed a sequence of exploratory analyses aimed at investigating the possible link between IR and (sub)cortical structure.

## 2 Materials and Methods

### 2.1 Participants

Patients with T2DM were recruited from the outpatients and inpatients of our hospital from October 2021 to October 2021. The diagnosis of T2DM was confirmed using various criteria, including self‐reported diabetes that had been previously diagnosed by an endocrinologist, current utilization of antidiabetic agents, fasting blood glucose (FBG) ≥7.0 mmol/L, hemoglobin A1c (HbA1c) ≥6.5%, or 2‐h postload glucose ≥11.1 mmol/L. The criteria adhere to the guidelines proposed by the American Diabetes Association (ADA) (American Diabetes Association 2021). The healthy controls (HCs) were individuals deemed to be in health after undergoing physical examinations. Inclusion criteria for all subjects were right‐handed and in the age range of 30–65 years.

The exclusion criteria for all participants were as follows: (1) other types of DM; (2) organic central nervous system disease; (3) history of severe head trauma; (4) major depression or other mental and psychiatric disorder; (5) major medical illness (cancer, anemia, or thyroid dysfunction); (6) alcoholism, tobacco addiction, long‐term drug use, or drug dependence; (7) obvious visual or hearing impairments; and (8) contraindications for MRI scanning. Finally, a total of 121 subjects, made up of 48 healthy subjects and 73 Type 2 diabetes patients, were enrolled.

### 2.2 Clinical Measurements and IR

We recorded all subjects’ height, weight, BMI ([weight in kg]/[height in m]^2^), arterial blood pressure, HbA1c, and FBG. Diabetes duration was determined by relying on the self‐reported beginning time. Serum markers of IR in T2DM patients after glucose provocation were assessed using the oral glucose tolerance test (OGTT). The OGTT, commonly utilized in clinical settings, can mimic glucose and insulin dynamics of physiological conditions (Park, Gautier, and Chon 2021). Following a minimum of ten hours of fasting overnight, participants underwent blood sampling for glucose and insulin at the designated time intervals: fasting at *t* = 0 immediately before the glucose challenge, followed by *t* + 30, *t* + 60, and *t* + 120 min after the glucose challenge with 75 g of an oral glucose solution. IR was determined by homeostatic model assessment of IR (HOMA‐IR) measures and the Matsuda Index. HOMA‐IR was calculated using the method: HOMA‐IR = [fasting insulin (µIU/mL)] × [fasting glucose (mmol/L)]/22.5. Calculation of the Matsuda Index founded on OGTT: Matsuda = 10,000 (*G*_0_ × *I*_0_ × *G*_mean_ × *I*_mean_)^1/2^ where *G*_0_ represents fasting glucose, *I*_0_ represents fasting insulin, *G*_mean_ stands for average serum glucose throughout the entire OGTT period, and *I*_mean_ stands for mean insulin throughout the entire OGTT period (Matsuda and DeFronzo 1999). A higher HOMA‐IR measure indicates a higher level of IR, and the opposite is true for the Matsuda Index.

### 2.3 Cognitive Tests

Preceding the acquisition of MRI images, all individuals were subjected to a comprehensive set of cognitive function assessments, including the Mini‐Mental State Examination (MMSE) (Shim et al. 2017), Montreal Cognitive Assessment (MoCA) (Li et al. 2018), Grooved Pegboard Test (GPT) (Tolle et al. 2019), Auditory Verbal Learning Test (AVLT) (Zhao et al. 2015), Digit Symbol Substitution Test (DSST) (Jaeger 2018), and Trail Making Test‐A (TMT‐A) (Mateen et al. 2018). It took about one hour for each subject to carry out all tests in a predetermined sequence.

### 2.4 MRI Acquisition

The MRI data were collected using the Siemens MAGNETOM Prisma 3.0 Tesla MRI scanner equipped with a 64‐channel head coil. During the acquisition of MRI images, the subjects maintained closed eyes and remained in a state of wakefulness. Cushions are employed to reduce the motion of the head, whereas earplugs are utilized to mitigate the impact of noise. Conventional brain axial T1WI and T2‐FLAIR sequences were obtained from all participants to exclude the organic brain lesions, including trauma, hemorrhage, cerebral infarction, and space‐occupying lesions. The parameters of the 3D T1WI sequence for brain structure analysis are as follows: inversion time = 1100 ms, repetition time = 2530 ms, echo time = 2.98 ms, flip angle = 7°, field of view = 256 × 256 mm^2^, slice thickness = 1.0 mm, number of slices = 192, voxel size = 1.0 × 1.0 × 1.0 mm^3^.

### 2.5 Brain MRI Analysis

The image‐processing pipeline provided by FreeSurfer (version 6.0.0, http://surfer.nmr.mgh.harvard.edu) was employed to perform the segmentation of bilateral cortical areas and subcortical structures, following the Desikan–Killiany atlas (Desikan et al. 2006). The bilateral cerebral cortex is divided into 68 subfields. The volume of subcortical brain structures and the mean thickness of cortical regions were extracted for each subject. Two skilled radiologists verified the accuracy of the deep subcortical gray matter structure segmentation and ruled out patients with neurological conditions. The reported sample size relies on whether anatomical data adhered to quality control standards, including subcortical nuclei volume and cortical thickness.

### 2.6 Statistical Analysis

The statistical analysis was completed using the Statistical Package for the Social Sciences (IBM, SPSS, version 26). Employ the Kolmogorov–Smirnov test to assess whether the data follow a normal distribution. For continuous variables, if they met normal distribution, they were assessed by independent two‐sample *t*‐tests and expressed as means and standard deviations. If not, they were assessed by the Mann–Whitney test and shown as medians and quartiles. The ratio data were determined using the chi‐squared test. The level of significance was established as *p* < 0.05.

Use analysis of covariance (ANCOVA) to investigate variations in subcortical brain volume and cortical thickness as a relation to T2DM, covarying for gender, age, education, and BMI. The scaling of head size is observed to have a direct relationship with volume, whereas thickness does not exhibit a similar correlation (Barnes et al. 2010). So estimated total intracranial volume (eTIV) was included as a covariate for subcortical brain volume. The Benjamini–Hochberg false discovery rate (FDR) correction was employed in order to address the issue of multiple comparisons, adjusting significance thresholds for thickness and volume analyses. If the FDR‐corrected *p* value of the outcome was less than 0.05, it was considered significant.

Within T2DM patients, we employed Spearman partial correlations to conduct exploratory analysis investigating relationships between HOMA‐IR, Matsuda Index, and brain structure, controlling for gender, age, BMI, and education. The eTIV was also incorporated as a covariate in our analysis of the brain volume. The outcomes were not adjusted for multiple comparisons, as the correlations were exploratory.

## 3 Results

### 3.1 Clinical Data and Neuropsychological Tests

Table 1 presents the demographic, clinical, and neuropsychological assessments of individuals with T2DM and healthy participants. The groups did not differ significantly in terms of age, gender, BMI, or arterial blood pressure. Patients with Type 2 diabetes had higher education level, HbA1c, and FBG (*p* < 0.001). Additionally, patients with T2DM exhibited lower MMSE scores (*p* = 0.001). No notable disparities were observed between the two groups in the remaining neuropsychological assessments.

**TABLE 1:** Demographic and clinical data of all participants.

	*T2DM* (*n* = 73)	HCs (*n* = 48)	Statistics	*p* value
General and clinical data				
Age (years)	48.47 ± 7.61	47.77 ± 6.88	*t* = 0.510	0.611
Gender (male/female)	47/26	32/16	*χ* ^2^ = −0.067	0.796
BMI (kg/m^2^)	24.03 ± 3.04	24.51 ± 3.18	*t* = −0.839	0.403
Education (years)	12 (9, 15.5)	9 (8, 9)	*z* = −4.831	<0.001^*^
SBP (mmHg)	127.75 ± 13.82	131.79 ± 14.37	*t* = −1.548	0.124
DBP (mmHg)	84.84 ± 10.72	85.75 ± 9.84	*t* = −0.474	0.636
Duration (years)	3 (1, 7.5)	N/A	N/A	N/A
HbA1c (%)	7.50 (6.40, 9.45)	5.65 (5.43, 5.90)	*z* = −8.093	<0.001^*^
FBG (mmol/L)	8.19 (6.99, 10.09)	5.20 (4.90, 5.48)	*z* = −8.493	<0.001^*^
FINS (µIU/mL)	8.90 (5.59, 14.91)	N/A	N/A	N/A
HOMR‐IR	3.33 (1.86, 5.61)	N/A	N/A	N/A
Matsuda Index	3.17 (2.03, 4.80)	N/A	N/A	N/A
Cognitive tests				
MMSE	28 (27, 29)	29 (28, 30)	*z* = −3.355	0.001^*^
MoCA	27 (25, 28)	27 (26, 28)	*z* = −1.354	0.176
GPT (R)	70 (64.5, 79)	69 (61.25, 77.75)	*z* = −0.594	0.553
GPT (L)	75 (67.25, 88.5)	76 (70.25, 85.75)	*z* = −0.888	0.375
AVLT (immediate)	23 (18.5, 26)	22 (17.25, 26)	*z* = −0.523	0.601
AVLT (5 min)	9 (7, 10.5)	9 (6, 10)	*z* = −0.740	0.460
AVLT (20 min)	8 (7, 10.5)	8 (6, 10)	*z* = −0.743	0.458
DSST	44.67 ± 14.54	41 ± 10.54	*t* = 1.294	0.198
TMT‐A	42 (32, 54.5)	45.5 (31.25, 63.75)	*z* = −0.538	0.591

*Note*: Data are presented as N, median (Q1, Q3), and mean ± SD.

Abbreviations: AVLT, auditory verbal learning test; BMI, body mass index; DBP, diastolic blood pressure; DSST, digit symbol substitution test; FBG, fasting blood glucose; FINS, fasting insulin; GPT, grooved pegboard test; HbA1c, hemoglobin A1c; HC, healthy control group; HOMA‐IR, homeostatic model assessment of insulin resistance; MMSE, mini‐mental state examination; MoCA, Montreal cognitive assessment; N/A, not applicable; SBP, systolic blood pressure; T2DM, Type 2 diabetes mellitus group; TMT‐A, trail making test‐A.

* *p* < 0.05.

### 3.2 Subcortical Brain Volume

Results are shown in Table 2. No substantial dissimilarities in the volume of subcortical brain were discovered. The bilateral amygdala volume showed slight but not significant increases in T2DM patients.

**TABLE 2:** Group differences in subcortical brain volume and cortical thickness.

	T2DM (*n* = 73)	HCs (*n* = 48)	*F* value	*p* value	*q* value	Partial *η* ^2^
eTIV (L)	1.57 ± 0.13	1.61 ± 0.12	3.338	0.070		
Subcortical brain volume (mL)						
L thalamus	7.30 ± 0.71	7.70 ± 0.78	1.671	0.199	0.358	0.014
R thalamus	7.10 ± 0.61	7.43 ± 0.73	1.654	0.201	0.358	0.014
L caudate	3.29 ± 0.34	3.41 ± 0.31	1.076	0.302	0.423	0.009
R caudate	3.39 ± 0.40	3.55 ± 0.38	1.619	0.206	0.358	0.014
L putamen	4.49 ± 0.55	5.12 ± 0.46	3.040	0.084	0.278	0.026
R putamen	5.03 ± 0.51	5.23 ± 0.47	3.529	0.063	0.278	0.030
L pallidum	2.10 ± 0.23	2.18 ± 0.15	0.257	0.613	0.627	0.002
R pallidum	1.99 ± 0.19	2.09 ± 0.17	1.457	0.230	0.358	0.013
L hippocampus	4.10 ± 0.33	4.13 ± 0.37	0.238	0.627	0.627	0.002
R hippocampus	4.29 ± 0.35	4.40 ± 0.40	0.819	0.368	0.468	0.007
L amygdala	1.71 ± 0.19	1.67 ± 0.21	5.083	0.026	0.278	0.043
R amygdala	1.79 ± 0.17	1.78 ± 0.19	4.019	0.047	0.278	0.034
L accumbens	0.43 ± 0.07	0.43 ± 0.09	0.342	0.560	0.627	0.003
R accumbens	0.50 ± 0.06	0.53 ± 0.07	2.763	0.099	0.278	0.024
Cortical thickness (mm)						
L bankssts	2.52 ± 0.10	2.54 ± 0.14	0.055	0.815	0.866	<0.001
R bankssts	2.56 ± 0.12	2.61 ± 0.14	6.584	0.012	0.066	0.054
L caudal anterior cingulate gyrus	2.58 ± 0.22	2.63 ± 0.21	1.921	0.168	0.327	0.016
R caudal anterior cingulate gyrus	2.46 ± 0.20	2.51 ± 0.17	0.933	0.336	0.532	0.008
L caudal middle frontal gyrus	2.57 ± 0.12	2.61 ± 0.09	7.659	0.007	0.055	0.062
R caudal middle frontal gyrus	2.51 ± 0.11	2.57 ± 0.09	16.643	<0.001	0.006^*^	0.126
L cuneus	1.83 ± 0.10	1.87 ± 0.15	4.033	0.047	0.171	0.034
R cuneus	1.91 ± 0.11	1.92 ± 0.13	0.270	0.604	0.705	0.002
L entorhinal area	3.56 ± 0.26	3.51 ± 0.25	0.461	0.499	0.632	0.004
R entorhinal area	3.65 ± 0.30	3.73 ± 0.31	3.390	0.068	0.202	0.029
L fusiform gyrus	2.78 ± 0.11	2.79 ± 0.12	0.588	0.445	0.606	0.005
R fusiform gyrus	2.79 ± 0.11	2.80 ± 0.12	0.653	0.421	0.596	0.006
L inferior parietal lobe	2.44 ± 0.10	2.47 ± 0.12	2.302	0.132	0.280	0.020
R inferior parietal lobe	2.47 ± 0.10	2.50 ± 0.11	2.905	0.091	0.221	0.025
L inferior temporal gyrus	2.83 ± 0.12	2.85 ± 0.10	0.506	0.478	0.625	0.004
R inferior temporal gyrus	2.80 ± 0.11	2.83 ± 0.10	1.293	0.258	0.444	0.011
L isthmus cingulate gyrus	2.32 ± 0.18	2.34 ± 0.19	0.411	0.523	0.647	0.004
R isthmus cingulate gyrus	2.34 ± 0.17	2.33 ± 0.18	0.220	0.640	0.725	0.002
L lateral occipital gyrus	2.18 ± 0.10	2.20 ± 0.11	0.786	0.377	0.557	0.007
R lateral occipital gyrus	2.24 ± 0.10	2.28 ± 0.11	6.585	0.012	0.066	0.054
L lateral orbitofrontal gyrus	2.61 ± 0.10	2.65 ± 0.11	7.333	0.008	0.055	0.060
R lateral orbitofrontal gyrus	2.58 ± 0.12	2.63 ± 0.14	2.304	0.132	0.280	0.020
L lingual gyrus	1.97 ± 0.10	1.98 ± 0.10	0.043	0.835	0.874	<0.001
R lingual gyrus	2.00 ± 0.09	2.01 ± 0.09	0.988	0.322	0.522	0.009
L medial orbitofrontal gyrus	2.46 ± 0.12	2.50 ± 0.12	3.744	0.055	0.171	0.032
R medial orbitofrontal gyrus	2.42 ± 0.12	2.45 ± 0.12	2.357	0.128	0.632	0.020
L middle temporal gyrus	2.88 ± 0.12	2.89 ± 0.13	0.674	0.413	0.596	0.006
R middle temporal gyrus	2.87 ± 0.13	2.90 ± 0.13	1.785	0.184	0.348	0.015
L parahippocampal gyrus	2.72 ± 0.26	2.64 ± 0.27	1.081	0.301	0.499	0.009
R parahippocampal gyrus	2.68 ± 0.19	2.67 ± 0.23	0.453	0.502	0.632	0.004
L paracentral lobule	2.49 ± 0.13	2.49 ± 0.12	0.543	0.463	0.617	0.005
R paracentral lobule	2.54 ± 0.14	2.56 ± 0.12	2.196	0.141	0.291	0.019
L pars opercularis	2.57 ± 0.11	2.62 ± 0.11	7.628	0.007	0.055	0.062
R pars opercularis	2.56 ± 0.11	2.62 ± 0.11	9.479	0.003	0.040^*^	0.076
L pars orbitalis	2.63 ± 0.16	2.66 ± 0.18	1.274	0.261	0.444	0.011
R pars orbitalis	2.59 ± 0.16	2.64 ± 0.18	2.920	0.090	0.221	0.025
L pars triangularis	2.44 ± 0.12	2.45 ± 0.11	0.278	0.599	0.705	0.002
R pars triangularis	2.43 ± 0.11	2.46 ± 0.11	3.269	0.073	0.205	0.028
L pericalcarine	1.58 ± 0.13	1.64 ± 0.13	3.221	0.075	0.204	0.027
R pericalcarine	1.60 ± 0.11	1.65 ± 0.12	6.132	0.015	0.077	0.051
L postcentral gyrus	2.08 ± 0.09	2.11 ± 0.12	3.833	0.053	0.171	0.032
R postcentral gyrus	2.06 ± 0.09	2.08 ± 0.12	1.647	0.202	0.361	0.014
L posterior cingulate gyrus	2.48 ± 0.17	2.49 ± 0.12	0.106	0.756	0.805	0.001
R posterior cingulate gyrus	2.46 ± 0.13	2.46 ± 0.15	0.005	0.945	0.954	<0.001
L precentral gyrus	2.64 ± 0.12	2.70 ± 0.12	10.067	0.002	0.040^*^	0.080
R precentral gyrus	2.58 ± 0.14	2.61 ± 0.15	4.263	0.041	0.169	0.036
L precuneus	2.39 ± 0.10	2.43 ± 0.10	3.906	0.050	0.171	0.033
R precuneus	2.41 ± 0.10	2.44 ± 0.11	4.222	0.042	0.169	0.035
L rostral anterior cingulate gyrus	2.78 ± 0.18	2.79 ± 0.21	1.679	0.198	0.361	0.014
R rostral anterior cingulate gyrus	2.77 ± 0.21	2.78 ± 0.16	0.362	0.549	0.666	0.003
L rostral middle frontal gyrus	2.36 ± 0.09	2.40 ± 0.08	5.235	0.024	0.116	0.044
R rostral middle frontal gyrus	2.30 ± 0.09	2.33 ± 0.08	3.941	0.050	0.171	0.033
L superior frontal gyrus	2.74 ± 0.10	2.80 ± 0.09	9.217	0.003	0.040^*^	0.074
R superior frontal gyrus	2.68 ± 0.09	2.74 ± 0.10	12.389	0.001	0.021^*^	0.097
L superior parietal lobe	2.21 ± 0.10	2.25 ± 0.09	4.281	0.041	0.169	0.036
R superior parietal lobe	2.20 ± 0.10	2.24 ± 0.10	3.064	0.083	0.216	0.026
L superior temporal gyrus	2.76 ± 0.11	2.78 ± 0.15	0.587	0.445	0.606	0.005
R superior temporal gyrus	2.77 ± 0.13	2.79 ± 0.16	2.103	0.150	0.299	0.018
L supramarginal gyrus	2.53 ± 0.11	2.57 ± 0.12	7.348	0.008	0.055	0.060
R supramarginal gyrus	2.50 ± 0.11	2.54 ± 0.12	7.263	0.008	0.055	0.059
L frontal pole	2.71 ± 0.19	2.77 ± 0.28	0.888	0.348	0.538	0.008
R frontal pole	2.66 ± 0.21	2.74 ± 0.23	2.718	0.102	0.239	0.023
L temporal pole	3.65 ± 0.22	3.63 ± 0.25	0.003	0.954	0.954	<0.001
R temporal pole	3.73 ± 0.24	3.73 ± 0.27	0.128	0.722	0.791	0.001
L transverse temporal gyrus	2.32 ± 0.19	2.31 ± 0.20	0.259	0.612	0.705	0.002
R transverse temporal gyrus	2.36 ± 0.20	2.37 ± 0.18	0.797	0.374	0.557	0.007
L insula	2.90 ± 0.14	2.92 ± 0.15	0.177	0.675	0.753	0.002
R insula	2.97 ± 0.11	2.98 ± 0.16	0.021	0.885	0.912	<0.001

*Note*: Covariates included gender, age, education, BMI, and, for regional volume estimates, estimated total intracranial volume. L, left hemisphere; R, right hemisphere; bankssts, banks of the superior temporal sulcus. Correction adjusted *p* value (*q* value) <0.05 had statistical significance.

Abbreviations: BMI, body mass index; eTIV, estimated total intracranial volume; HC, healthy control; T2DM, Type 2 diabetes mellitus.

* Correction adjusted *p* value (*q* value) <0.05.

### 3.3 Cortical Thickness

Results are presented in Table 2. We found that cortical thickness in the T2DM group was reduced, particularly in the right pars opercularis, right caudal middle frontal gyrus, left precentral gyrus, and bilateral superior frontal gyrus. The thickness of the remaining brain regions did not reveal significant differences between the two groups.

### 3.4 Exploratory Brain‐IR Correlations

The exploratory correlations between brain structure and IR are presented in Table 3. In the T2DM group, the results of the partial correlation analysis indicate a negative correlation between the IR and the volume of the left putamen (*r* = −0.258, *p* = 0.034) and left hippocampus (*r* = −0.261, *p* = 0.031), as well as the thickness of the left pars orbitalis (*r* = −0.260, *p* = 0.031) and left pericalcarine (*r* = −0.345, *p* = 0.004). The Matsuda Index demonstrated a significant positive correlation with the thickness of the right entorhinal area (*r* = 0.245, *p* = 0.042) and right rostral anterior cingulate gyrus (*r* = 0.250, *p* = 0.038).

**TABLE 3:** Brain‐insulin resistance (IR) partial correlations in Type 2 diabetes mellitus (T2DM) group.

	HOMA‐IR	Matsuda index		HOMA‐IR	Matsuda index
Subcortical brain volume					
L thalamus	−0.072	−0.063	R thalamus	−0.040	−0.120
L caudate	−0.028	0.055	R caudate	0.055	0.027
L putamen	−0.258^*^	0.034	R putamen	−0.010	−0.005
L pallidum	−0.077	−0.096	R pallidum	−0.206	−0.029
L hippocampus	−0.261^*^	0.190	R hippocampus	−0.160	0.221
L amygdala	−0.204	0.171	R amygdala	−0.191	0.139
L accumbens	−0.203	0.195	R accumbens	−0.161	0.152
Cortical thickness					
L bankssts	−0.118	0.145	R bankssts	−0.090	0.081
L caudal anterior cingulate gyrus	0.105	−0.058	R caudal anterior cingulate gyrus	0.185	−0.142
L caudal middle frontal gyrus	0.054	−0.181	R caudal middle frontal gyrus	0.006	−0.230
L cuneus	−0.144	0.002	R cuneus	−0.030	−0.147
L entorhinal area	−0.086	0.143	R entorhinal area	−0.085	0.245^*^
L fusiform gyrus	0.086	0.143	R fusiform gyrus	0.088	−0.067
L inferior parietal lobe	−0.101	−0.025	R inferior parietal lobe	0.068	−0.151
L inferior temporal gyrus	−0.032	−0.038	R inferior temporal gyrus	−0.094	0.083
L isthmus cingulate gyrus	−0.005	−0.052	R isthmus cingulate gyrus	−0.011	−0.075
L lateral occipital gyrus	−0.012	−0.155	R lateral occipital gyrus	−0.059	−0.034
L lateral orbitofrontal gyrus	−0.217	0.023	R lateral orbitofrontal gyrus	−0.050	0.004
L lingual gyrus	−0.133	0.097	R lingual gyrus	−0.147	−0.015
L medial orbitofrontal gyrus	0.129	−0.012	R medial orbitofrontal gyrus	−0.002	−0.069
L middle temporal gyrus	−0.174	0.074	R middle temporal gyrus	−0.080	−0.117
L parahippocampal gyrus	−0.178	0.109	R parahippocampal gyrus	−0.088	0.003
L paracentral lobule	0.039	−0.105	R paracentral lobule	−0.032	0.058
L pars opercularis	0.007	−0.068	R pars opercularis	−0.122	0.108
L pars orbitalis	−0.260^*^	0.057	R pars orbitalis	−0.081	0.030
L pars triangularis	−0.079	0.086	R pars triangularis	−0.116	−0.070
L pericalcarine	−0.345^*^	0.163	R pericalcarine	−0.177	0.159
L postcentral gyrus	0.157	−0.069	R postcentral gyrus	0.163	−0.011
L posterior cingulate gyrus	0.075	0.170	R posterior cingulate gyrus	−0.025	0.031
L precentral gyrus	0.062	−0.108	R precentral gyrus	−0.062	−0.062
L precuneus	0.017	−0.085	R precuneus	0.024	−0.042
L rostral anterior cingulate gyrus	−0.016	0.060	R rostral anterior cingulate gyrus	−0.178	0.250^*^
L rostral middle frontal gyrus	−0.176	0.001	R rostral middle frontal gyrus	−0.052	0.037
L superior frontal gyrus	−0.002	−0.148	R superior frontal gyrus	−0.025	−0.184
L superior parietal lobe	0.145	−0.062	R superior parietal lobe	0.110	−0.158
L superior temporal gyrus	0.079	−0.022	R superior temporal gyrus	−0.041	0.038
L supramarginal gyrus	0.054	−0.17	R supramarginal gyrus	0.064	−0.134
L frontal pole	<0.001	0.033	R frontal pole	−0.132	−0.024
L temporal pole	−0.109	0.054	R temporal pole	−0.136	0.123
L transverse temporal gyrus	0.116	−0.045	R transverse temporal gyrus	0.165	−0.054
L insula	−0.058	0.097	R insula	0.050	0.028

*Note*: Partial correlations controlled for gender, age, education, BMI, and, for volume analyses, total intracranial volume. L, left hemisphere; R, right hemisphere.

Abbreviations: BMI, body mass index; FDR, false discovery rate; HOMA‐IR, homeostatic model assessment of insulin resistance.

* *p* < 0.05 (correlations were not FDR‐corrected).

## 4 Discussion

In this study, the structure of subcortical and cortical gray matter was analyzed in the T2DM patients and compared to normoglycemic HCs. Lower thickness in the right pars opercularis, right caudal middle frontal gyrus, left precentral gyrus, and bilateral superior frontal gyrus was observed in T2DM group. Additional correlation study conducted among individuals with T2DM revealed a significant relationship between modified brain structure and IR, which cannot be disregarded.

Lower scores on the MMSE were observed in patients with T2DM compared to HCs. Prior research has discovered that MMSE scores would be influenced by education, with higher education levels leading to higher MMSE scores (Keskįnoğlu et al. 2009). It is notable that the T2DM group with higher education levels compared to HCs group had lower MMSE scores in this study. It hints at the fact that Type 2 diabetes predisposes to cognitive decline, which is in line with prior research findings (Callisaya et al. 2018; Zilliox et al. 2016).

We did not observe significant variations in subcortical brain volume between T2DM patients and HCs. However, some investigations have reported alterations in hippocampus volumes in persons with T2DM (Moulton et al. 2015). Another study on T2DM and prediabetic patients found that the right putamen, left amygdala, and bilateral lateral hippocampus were demonstrated significant reduction (Cui et al. 2019). With respect to cortical thickness, we reported alterations in the thickness of the right caudal middle frontal gyrus, right pars opercularis, left precentral gyrus, and the bilateral superior frontal gyrus, which all belong to the frontal lobe. The results of this study support prior research that also identified abnormalities in T2DM patients in similar areas (Bernardes et al. 2018; Brundel et al. 2010; Moulton et al. 2015). Many of the previous studies, however, demonstrated outcomes that were spatially more extensive than those obtained from our research. Methodological and/or clinical factors may explain this. Regarding methodology, we extracted the volume and thickness of anatomically defined brain subregions. A whole‐brain voxel‐based morphometry or empirically defined region of interest method may have produced varied outcomes (Moulton et al. 2015). In addition, we statistically controlled for covariates known to correlate with changes in brain anatomy. Clinically, due to the limited sample size, we did not analyze the use of various medication and cognitive stages of patients in depth. The frontal lobe comprises different regions that are responsible for specific brain processes, including the frontal eye fields, higher order cognitive functions, motor area, and supplementary motor areas. The classification of higher order cognitive functions can be further refined into the following categories: executive function, energization, emotional/behavioral control and decision‐making, and metacognition or consciousness (Stuss 2008). Frontal lobe functions, such as executive functioning, working memory, and attention, have been the subject of investigation (Henri‐Bhargava, Stuss, and Freedman 2018). The development and degeneration of brain tissue are significantly characterized by alterations in cortical thickness, which plays a pivotal role in several neurological conditions, including neurodegenerative illnesses. The significant decrease of cortical thickness in the frontal lobe might be a leading factor of cognitive impairment (Cheng et al. 2018).

Within patients with T2DM, it was observed that decreased thickness or volume of certain brain regions was correlated with increased severity of IR. Modern neuroimaging research has unveiled a notable insulin‐induced brain response, predominantly in the fusiform gyrus, insular cortex, prefrontal cortex, hypothalamus, striatum, and hippocampus (Kullmann et al. 2016). An animal experiment using 125I‐labeled insulin to evaluate insulin receptors in the rat brain uncovered their extensive distribution throughout the olfactory bulb, cerebral cortex, hypothalamus, hippocampus, amygdala, and septum (Hill et al. 1986). The brain regions significantly correlated with IR in our study coincide with the above findings. Most studies about T2DM attributed the phenomenon of alterations in volume to glycemic dysregulation, dyslipidemia, and accompanying vasculopathy (Cui et al. 2022). However, the exact mechanism remains unclear. Our study provides insights for future research in this field.

The hippocampus and neighboring entorhinal cortex are particularly important regions in the temporal lobe for cognition and memory formation (Gerlei et al. 2021; Lisman et al. 2017). The lateral prefrontal cortex, encompassing the pars orbitalis and rostral anterior cingulate gyrus (Fuster 2001), is of the utmost importance in cognitive function, such as the inhibitory control of eating behavior (Hare, Camerer, and Rangel 2009). The putamen constitutes a component of the striatum, which is integral to the cortico‐striatal‐thalamic circuits responsible for regulating reward learning and motivation. These processes are commonly impaired during periods of depression (Pizzagalli 2014). As the severity of IR increases, the volume and thickness of these areas reduce. We posit that IR impacts alterations in brain structure, which consequently leads to brain dysfunction.

The findings of our investigation should be interpreted within the framework of specific constraints. First, the research design employed in this study is cross‐sectional, and it is worth noting that the sample size utilized is relatively small. Therefore, our ability to provide answers on causal relationships between brain structure and IR is limited. The limited sample size may potentially hinder the extrapolation of our findings to broader, more heterogeneous populations. Second, we did not exclude the effect of insulin‐sensitizing drugs such as metformin or thiazolidinediones on the IR assessment. Insulin‐sensitizing drugs not only improve peripheral IR but also show some potential in the therapeutic management of Alzheimer's disease (Yang 2022). Failure to take medication into account may have affected the reliability of our results. Finally, HOMA‐IR and the Matsuda Index are not the gold standard for evaluating IR. More precise measurements, such as hyperinsulinemic‐euglycemic clamp, may provide a more accurate assessment, but these are invasive and labor‐intensive. Therefore, it is imperative to develop a low‐cost, noninvasive approach that accurately reflects the physiology of insulin and glucose metabolism (Park, Gautier, and Chon et al. 2021).

## 5 Conclusion

This study indicated the thickness of several cortical subfields decreased and alterations in brain structure were correlated with IR in T2DM patients. IR might play a pivotal role in structural brain alterations in T2DM individuals. We hypothesize that IR affects brain structure, which in turn leads to a string of diabetes‐related brain damage. Additional clinical and fundamental research is necessary to validate the findings obtained from the present investigation. Future studies could focus on pathophysiological mechanisms, ultimately to mitigate or preempt the adverse consequences of T2DM on brain.

## Author Contributions

Conception and study design: Zidong Cao and Limin Ge. Data collection or acquisition: Zidong Cao and Weiye Lu. Statistical analysis: Kui Zhao, Yuna Chen, and Zhizhong Sun. Interpretation of results: Wenbin Qiu, Xiaomei Yue, and Yifan Li. Drafting the manuscript work or revising it critically for important intellectual content: Zidong Cao and Shijun Qiu. All authors approve the final version to be published and agree to be accountable for the integrity and accuracy of all aspects of the work.

## Ethics Statement

The studies involving human participants were reviewed and approved by Medical Research Ethics Committee of Guangzhou University of Chinese Medicine (No. K2020‐115).

## Consent

The patients/participants provided their written informed consent to participate in this study.

## Conflicts of Interest

The authors declare no conflicts of interest.

### Peer Review

The peer review history for this article is available at https://publons.com/publon/10.1002/brb3.70055.

## Data Availability

The data that support the findings of this study are available from the corresponding author upon reasonable request.
